# Acute and Long COVID Intestinal Changes in an Experimental Model of Coronavirus in Mice

**DOI:** 10.3390/v16060832

**Published:** 2024-05-24

**Authors:** Hussain Hussain, Nila Elumalai, Natarajan Sampath, Nagarajarao Shamaladevi, Rima Hajjar, Brian Zachary Druyan, Amirah B. Rashed, Rajalakshmi Ramamoorthy, Norma S. Kenyon, Arumugam R. Jayakumar, Michael J. Paidas

**Affiliations:** 1 Department of Obstetrics, Gynecology and Reproductive Sciences, University of Miami Miller School of Medicine, Miami, FL 33136, USA; hussainhussainmd77@gmail.com (H.H.); nilanandhie@gmail.com (N.E.); rima.hajjar@jhsmiami.org (R.H.); bzd2@med.miami.edu (B.Z.D.); abr101@med.miami.edu (A.B.R.); rxr1310@med.miami.edu (R.R.); 2 Department of Internal Medicine, HCA Florida Kendall Hospital, Miami, FL 33175, USA; 3 School of Chemical and Biotechnology, SASTRA Deemed University, Thanjavur 613401, India; sams76@gmail.com; 4 Molecular Analytics, Miami, FL 33187, USA; nithumidhu2011@gmail.com; 5 Microbiology & Immunology and Biomedical Engineering, Diabetes Research Institute, University of Miami, Miami, FL 33136, USA; nkenyon@med.miami.edu; 6 Department of Biochemistry and Molecular Biology, The University of Miami Miller School of Medicine, Miami, FL 33136, USA

**Keywords:** intestine, fibrosis, infection, pili, goblet cell, long COVID, murine hepatitis virus-1

## Abstract

The COVID-19 pandemic, which emerged in early 2020, has had a profound and lasting impact on global health, resulting in over 7.0 million deaths and persistent challenges. In addition to acute concerns, there is growing attention being given to the long COVID health consequences for survivors of COVID-19 with documented cases of cardiovascular abnormalities, liver disturbances, lung complications, kidney issues, and noticeable cognitive deficits. Recent studies have investigated the physiological changes in various organs following prolonged exposure to murine hepatitis virus-1 (MHV-1), a coronavirus, in mouse models. One significant finding relates to the effects on the gastrointestinal tract, an area previously understudied regarding the long-lasting effects of COVID-19. This research sheds light on important observations in the intestines during both the acute and the prolonged phases following MHV-1 infection, which parallel specific changes seen in humans after exposure to SARS-CoV-2. Our study investigates the histopathological alterations in the small intestine following MHV-1 infection in murine models, revealing significant changes reminiscent of inflammatory bowel disease (IBD), celiac disease. Notable findings include mucosal inflammation, lymphoid hyperplasia, goblet cell hyperplasia, and immune cell infiltration, mirroring pathological features observed in IBD. Additionally, MHV-1 infection induces villous atrophy, altered epithelial integrity, and inflammatory responses akin to celiac disease and IBD. SPIKENET (SPK) treatment effectively mitigates intestinal damage caused by MHV-1 infection, restoring tissue architecture and ameliorating inflammatory responses. Furthermore, investigation into long COVID reveals intricate inflammatory profiles, highlighting the potential of SPK to modulate intestinal responses and restore tissue homeostasis. Understanding these histopathological alterations provides valuable insights into the pathogenesis of COVID-induced gastrointestinal complications and informs the development of targeted therapeutic strategies.

## 1. Introduction

The enduring global ramifications of the COVID-19 pandemic persist, marked by widespread morbidity and mortality. By 12 January 2023, the cumulative death toll had surpassed 7.7 million since the pandemic’s inception in early 2020, coupled with a daily surge of over 39,000 new cases worldwide [1]. Encouragingly, out of 775 million individuals affected, 767 million have successfully recuperated from mild to severe SARS-CoV-2 infections [1]. Despite collaborative global endeavors towards vaccine development to mitigate mortality rates, there remains a conspicuous absence of specific therapeutic interventions for viral infection [1,2,3,4].

In addition to the acute manifestations of COVID-19, survivors contend with persistent health challenges, including disrupted sleep patterns, osteoporosis, subfertility, exacerbated diabetes, fatigue, and complications affecting various bodily systems such as musculoskeletal, cardiovascular, gastrointestinal, pulmonary, neurologic, and urologic systems [1,2,5,6,7,8]. The intricate interplay between genetic mutations of SARS-CoV-2 and the spectrum of documented post-infection complications poses a significant challenge for scientists in elucidating the precise pathophysiological mechanisms. Furthermore, the progression of disease pathogenesis, spanning from weeks to months, demonstrates indications of multiple organ dysfunction or failure occurring in one or more instances following post-acute SARS-CoV-2 infection—referred to as post-acute sequelae of SARS-CoV-2 infection, whether presenting with or without apparent symptoms and behavioral changes.

Research findings indicate the emergence of a clinical syndrome akin to severe acute respiratory syndrome (SARS) in mice infected with murine hepatitis virus-1 (MHV-1), presenting with a notably high mortality rate [9,10,11,12,13,14,15,16,17,18]. These mice exhibit marked lung injury, resulting in mortality rates ranging from 40–60% between days 7 and 12 post-infection [18]. Upon post-mortem examination, severe interstitial pneumonitis is evident in the lungs, characterized by interstitial inflammatory reactions and substantial infiltration of lymphocytes and macrophages [16,18]. Additionally, investigations into the livers of MHV-1-infected A/J mice unveil evidence of severe hepatic congestion, resembling observations in humans infected with SARS-CoV-2 [16,18].

In conjunction with multi-organ dysfunction, alterations in the intestine linked to COVID-19 have been confirmed during the acute stages post-infection. The intestine exhibits a spectrum of manifestations that can vary significantly among individuals.

Considering the shared genus among MHV, severe acute respiratory syndrome coronavirus, and SARS-CoV-2, insights from MHV-1 could potentially yield mechanistic comprehension of SARS-CoV-2 infection in humans [1,2,3]. While notable parallels exist between MHV-1 in murine hosts and SARS-CoV-2 in humans, encompassing specific pathogenic characteristics, discernible differences also manifest. For instance, variations in viral binding receptors are evident, with SARS-CoV-2 engaging angiotensin-converting enzyme-2 (ACE2) while MHV-1 interacts with carcinoembryonic antigen-related cell adhesion molecule 1 (CEACAM1) [15,16,18,19]. Further distinctions encompass the proteolytic cleavage of four essential amino acids at the S1/S2 site of the SARS-CoV-2 spike protein [19,20,21]. Nevertheless, the noted similarities outweigh the differences. Significantly, the delineation of pathological and functional alterations in the MHV-1 murine model of COVID-19 underscores a substantial level of analogy to humans afflicted by SARS-CoV-2 infection [15,16,18].

In this investigation, we aimed to explore the correlation between acute and enduring intestinal modifications in COVID-19. Additionally, we sought to assess whether the inhibition of viral entry through the utilization of a recently identified 15-amino-acid synthetic peptide, SPIKENET (SPK), which effectively impedes the binding of Spike glycoprotein-1 with host receptors and exhibits potent anti-inflammatory properties in response to severe inflammatory stimuli, can mitigate or prevent intestinal alterations.

## 2. Materials and Methods

### 2.1. Mice 

We used 8-week-old female A/J mice weighing 22–24 g each. These mice were purchased from Jackson Laboratories (Bar Harbor, ME, USA) and were kept in cages at the University of Miami Miller School of Medicine animal isolation facility. The animals were fed a standard lab chow diet (Envigo 2918 irradiated, Teklad diet, Dublin, VA, USA) and provided with water ad libitum (autoclaved tap water). The study was performed according to the guidelines of the University of Miami Institutional Animal Care and Use Committee (IACUC protocol number 20–131 LF/Renewed protocol number 20–162). Experimental groups: In the acute investigation, these mice were divided into 4 groups: MHV-1 infection alone (*n* = 16), healthy control (*n* = 7), SPK alone (*n* = 5), and SPK-treated mice (*n* = 5). For the long COVID study, we investigated 12 mice (4 MHV-1 infection, 4 healthy control, 4 SPK treated group).

### 2.2. Viral (MHV-1) Inoculation and SPIKENET 

MHV-1 treatment was procured from the American Type Culture Collection (ATCC, cat# VR 261, Manassas, VA, USA). Mice were stratified into four groups: (1) a healthy control cohort, (2) a group inoculated with MHV-1 virus, (3) a group inoculated by SPK alone, and (4) a group inoculated with MHV-1 virus and subsequently treated with SPK. MHV-1 viral inoculation was executed following established protocols [11,15,16,18,19] involving intranasal administration of 5000 PFU MHV-1 to groups 2 and 4 with vigilant monitoring to ensure adequate inhalation of the virus. Mice in group 4, receiving MHV-1 inoculation, were further treated with 5 mg/kg body weight of SPIKENET (SPK), as previously outlined (3 doses of 5 mg/kg given every alternate day from day 2, i.e., 2, 4, and 6 days post-MHV-1) [14,15,16,18,19]. 

### 2.3. Intestine Tissues Collection and Storage

The intestinal tissue of the mice was harvested, with fecal matter carefully removed before fixation in 10% formalin. For acute studies, this process occurred 7 days post-infection, while for long COVID investigations, it took place 12 months post-infection. Subsequently, the specimens were embedded in paraffin and sectioned into 10 µm thick slices using an ultra-thin semiautomatic microtome. This cutting procedure was conducted utilizing the Histoscore auto-cut automated rotary microtome from Leica Biosystems Inc., Buffalo Grove, IL, USA, following processing through the Histoscore PELORIS 3 Premium Tissue Processing System.

### 2.4. Histological Staining 

Histological staining of the mice intestine with hematoxylin and eosin (H&E) was conducted using the following materials and reagents: SelecTech hematoxylin and eosin staining system (consisting of Hematoxylin 560, Blue Buffer 8, Define, Alcoholic Eosin Y 515, and Eosin Trichrome 515) (Leica Biosystems, Cat# 3801570/3801615, IL, USA), formalin-fixed paraffin-embedded mice intestinal tissue sections, xylene (C8H10, CAS RN 106-42-3, Fisher Scientific, Hampton, NH, USA), absolute ethanol (E7023, Sigma-Aldrich, St. Louis, MO, USA), 95% ethanol, 70% ethanol, distilled water, microscope slides (Epredia™ Premium Microscope Slides in Tropical Packaging, Cat# 22-339-411, Portsmouth, NH, USA), and coverslips (Gold Seal^®^ Cover Glass (Cat# 6376501, Electron Microscopy Sciences, Hatfield, PA, USA) [22]. Initially, deparaffinization was achieved through the following steps: the slides were immersed in xylene for 5 min, followed by absolute ethanol for 3 min, then placed in 95% ethanol for 2 min, and subsequently in 70% ethanol for 2 min [22]. The tissue sections were then rinsed with distilled water. Hematoxylin staining was performed by submerging the slides in hematoxylin solution for 5–10 min, followed by rinsing with distilled water to remove excess stain and differentiation with 1% acid alcohol until sections turned blue. The slides were then rinsed again in distilled water [22]. Subsequently, eosin staining was carried out by exposing the slides to eosin Y solution for 2–3 min, followed by rinsing with distilled water [22]. Dehydration of the slides was accomplished by sequentially immersing them in 70%, 95%, and absolute ethanol, followed by placement in xylene for 5 min. Finally, the slides were mounted in mounting media, and coverslips were applied [22].

## 3. Results

While primarily recognized as a respiratory illness, emerging evidence suggests that SARS-CoV-2 infection can also impact the gastrointestinal tract, with notable changes occurring in the small intestine. Diverse histological alterations were discerned during our scrutiny of MHV-1 infection within the murine intestinal, probing into acute, long COVID, and SPK treatments as well as SPK alone (SPK did not display any changes in the control group, which was identical to the healthy group (figure is not shown)). Whereas the precise mechanisms by which SARS-CoV-2 affects the small intestine remain under investigation, several hypotheses have been proposed. These include direct viral invasion of enterocytes expressing the angiotensin-converting enzyme 2 (ACE2) receptor, dysregulation of the gut microbiota, immune-mediated damage, and systemic effects of cytokine release.

**Figure 1:**
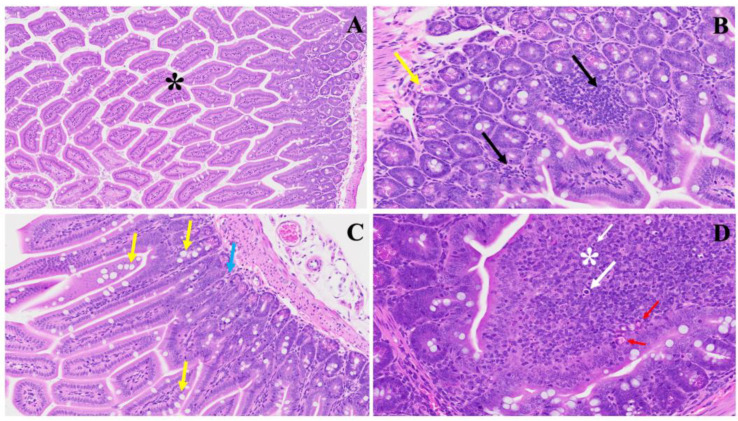
This illustrates the histopathological changes observed in acute extensive intestinal inflammation in the MHV-1 model in small and large intestines. In Panel (**A**), the typical architecture of the large intestine is depicted, with intact villi highlighted by a star. Panel (**B**) demonstrates colonic mucosal inflammation and lymphoid hyperplasia, indicated by black arrows, accompanied by microthrombi marked by yellow arrows. In Panel (**C**), hyperplasia of goblet cells scattered throughout the colonic mucosa is shown (yellow arrows), along with various inflammatory cells within the lamina propria (blue arrow). Panel (**D**) reveals diffuse proliferation of lymphoid tissue (white star), microbleeding (red arrow), and melanocytosis (white arrows) in the colonic mucosa. These findings provide insight into the pathological features associated with acute significant intestinal changes in the MHV-1 model. (H&E, original magnification 66× (**A**–**D**)). MHV-1 infection alone (*n* = 16), healthy control (*n* = 7), and SPK-treated mice (*n* = 5).

The histopathological findings in Figure 1 shed light on the acute significant intestinal changes observed in the MHV-1 model and their potential relevance to inflammatory bowel disease (IBD) [23,24]. The model presents notable alterations in colonic architecture, including mucosal inflammation, lymphoid hyperplasia, and microthrombus formation, as highlighted in Panel (B). These features resemble the inflammatory processes commonly observed in IBD, where acute, chronic inflammation in the gastrointestinal tract leads to tissue damage and dysfunction. In (C), we noticed hyperplasia of goblet cells scattered throughout the colonic mucosa, indicative of an adaptive response to mucosal injury. This finding aligns with the increased mucus production often observed in IBD [24], which protects against luminal insults. Moreover, various inflammatory cells within the lamina propria, as depicted by the blue arrow, suggest an active immune response in the affected tissue. We observed diffuse proliferation of lymphoid tissue, microbleeding, and melanocytosis in the colonic mucosa (D), further indicating ongoing inflammation and tissue damage. These pathological features resemble the characteristic histological changes seen in IBD, such as crypt distortion, epithelial ulceration, and infiltration of immune cells into the mucosa and submucosa.

**Figure 2:**
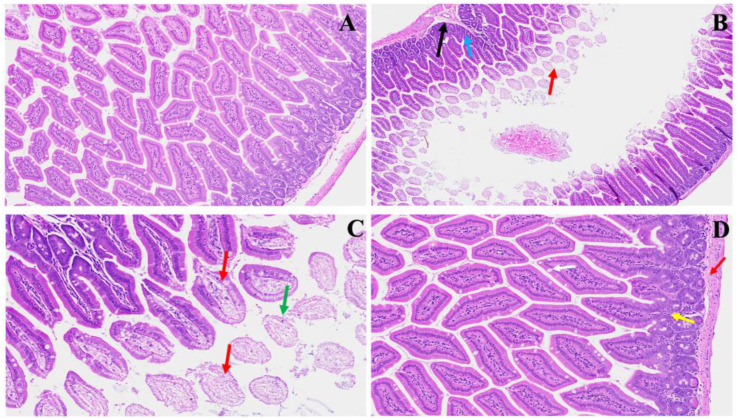
This shows acute small and large intestinal changes induced by MHV-1 infection. In Panel (**A**), a histological section displays the typical regular colonic layers. Panel (**B**) exhibits early manifestations of disease, with evident mucosal layer sloughing (red arrow), concomitant inflammatory alterations in the muscularis mucosa layer (black arrow), and overall inflammation (blue arrow). Panel (**C**) depicts diverse stages of villus degeneration (red arrows) alongside the presence of crypt apoptotic bodies (green arrow). In Panel (**D**), regenerative responses during the acute phase of SPK treatment are evident, including the reconstruction of the muscularis mucosa layer (red arrow), normalization of goblet cells (white arrow), restoration of crypt and lamina propria anatomy (yellow arrow), and mitigation of inflammatory changes. (H&E, original magnification 66× (**A**–**D**)). MHV-1 infection alone (*n* = 16), healthy control (*n* = 7), and SPK-treated mice (*n* = 5).

We found significant changes in this model. These alterations could parallel COVID-induced colonic issues, as shown in Figure 2. Panel B emphasizes early pathological features following MHV-1 infection, characterized by visible mucosal layer sloughing (red arrow) accompanied by inflammatory alterations within the muscularis mucosa layer (black arrow) and generalized inflammation (blue arrow). These findings resonate with reports of COVID-induced colonic manifestations, such as mucosal injury and inflammatory responses. Panel C demonstrates the progression of villus degeneration (red arrows) alongside the presence of crypt apoptotic bodies (green arrow), suggesting a continuum of tissue damage reminiscent of COVID-associated gastrointestinal complications. In Panel D, regenerative responses during the acute phase, particularly following SPK treatment, are evident. These include the reconstruction of the muscularis mucosa layer (red arrow), normalization of goblet cells (white arrow), restoration of crypt and lamina propria anatomy (yellow arrow), and mitigation of inflammatory changes. Understanding these histopathological alterations contributes to elucidating the pathogenesis of MHV-1 infection and provides insights into potential therapeutic strategies for mitigating COVID-induced colonic issues. 

**Figure 3:**
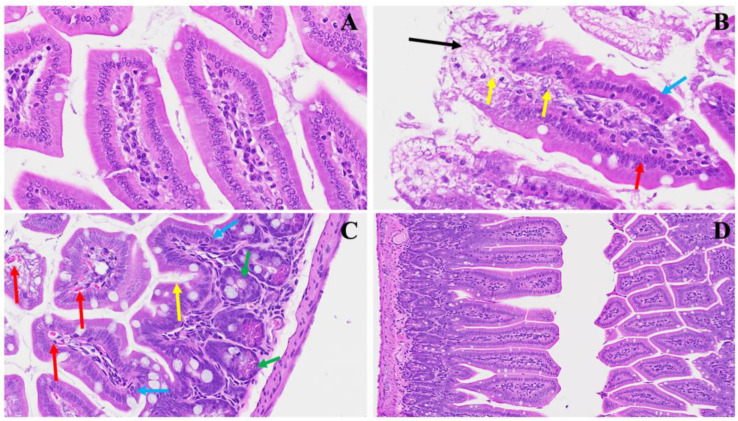
This portrays the acute small intestinal changes ensuing from MHV-1 infection. In Panel (**A**), a depiction of normal crypt morphology with intact goblet cells is observed. Panel (**B**) showcases several mitotic figures within the villi (yellow arrows) alongside sloughing villi (black arrow) and the destruction of enterocytes, representing the simple columnar epithelium (red arrow) accompanied by edema surrounding enteroendocrine cells (blue arrow). Panel (**C**) further elucidates the presence of edema surrounding enteroendocrine cells (blue arrow), dying Paneth cells (green arrow), increased mucus secretion (yellow arrow), and invasion of red blood cells (red arrows). Panel (**D**) demonstrates the restoration of regular histopathological changes following SPK administration characterized by reduced sloughing and normalization of goblet cells. These observations offer insights into the dynamic alterations occurring in the small intestine during MHV-1 infection and hint at the potential therapeutic benefits of SPK treatment. (H&E, original magnification 66× (**A**–**C**) and 22× (**D**)). MHV-1 infection alone (*n* = 16), healthy control (*n* = 7), and SPK-treated mice (*n* = 5).

We also examined the small intestinal changes post MHV-1 infection, with implications for potential therapeutic interventions such as SPK treatment. Figure 3, Panel A serves as a reference point, depicting the typical crypt morphology with intact goblet cells. As shown in panel B, along with several mitotic figures within the villi (yellow arrows), characteristic pathological features of MHV-1 infection emerge, including sloughing villi (black arrow) and the destruction of enterocytes, representing the simple columnar epithelium (red arrow). Notably, edema surrounding enteroendocrine cells (blue arrow) mirrors histopathological changes observed in conditions like celiac disease, where disruption of the epithelial barrier and villous atrophy are prominent features. Findings reminiscent of inflammatory bowel disease (IBD) [23,24], such as altered epithelial integrity and increased enterocyte turnover, are apparent.

Panel C offers further insight into the pathological cascade, unveiling pronounced edema surrounding enteroendocrine cells (blue arrow), the presence of dying Paneth cells (green arrow), increased mucus secretion (yellow arrow), and invasion of red blood cells (red arrows). These findings corroborate the multifaceted nature of MHV-1-induced intestinal damage and underscore similarities with the histopathological alterations in celiac disease and IBD, including Paneth cell abnormalities and altered mucin secretion, contributing to mucosal inflammation and barrier dysfunction.

Panel D highlights the potential therapeutic benefits of SPK administration, showcasing the restoration of regular histopathological changes characterized by reduced sloughing and normalization of goblet cells. These observations deepen our understanding of the dynamic alterations occurring in the small intestine during MHV-1 infection and underscore the relevance of studying viral-induced intestinal pathology in the context of celiac disease and IBD [23,24,25]. 

**Figure 4:**
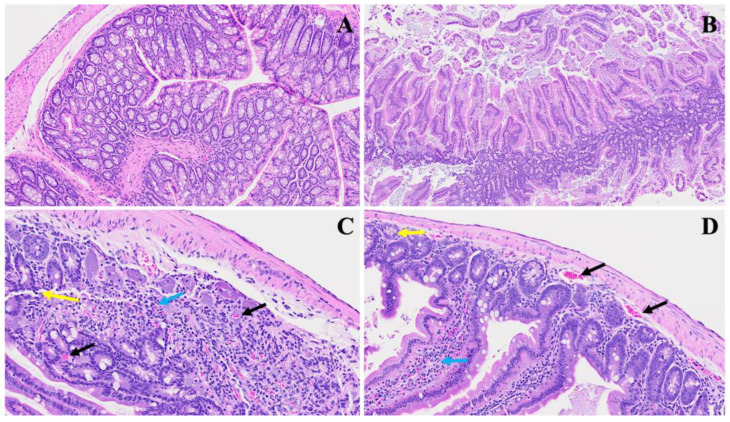
Representation of acute small bowel damage induced by MHV-1 infection. Panel (**A**) demonstrates normal small bowel histology, while Panel (**B**) depicts severe inflammatory changes penetrating all layers of the small intestine, including papillary necrosis, apoptosis, and an inflamed lamina propria. Panel (**C**) highlights the infiltration of immune cells, with neutrophils (yellow arrow) and lymphocytes (blue arrow) evident alongside observable microthrombi (black arrows). Panel (**D**) further illustrates microthrombi (black arrows), dying crypts (yellow arrows), and pronounced villi inflammation (blue arrows). (H&E, original magnification 22× (**A**–**D**)). MHV-1 infection alone (*n* = 16), healthy control (*n* = 7), and SPK-treated mice (*n* = 5).

Figure 4 shows various intestinal damage induced by MHV-1 infection in an acute setting. There are severe inflammatory changes evident, penetrating all layers of the small intestine in Panel B. Papillary necrosis, apoptosis, and inflammation of the lamina propria indicate significant tissue damage, reminiscent of findings reported in COVID-19 patients with gastrointestinal symptoms as well as with IBD [24]. In Panel C, we observe the infiltration of immune cells, including neutrophils and lymphocytes, into the small intestinal tissue. These cells play crucial roles in the immune response against viral infections but can also contribute to tissue damage when dysregulated. Additionally, microthrombi, indicated by black arrows, highlight the potential involvement of coagulation abnormalities in small bowel pathology during viral infections. Meanwhile, Panel D further emphasizes the presence of microthrombi, dying crypts, and pronounced inflammation of the villi. The disruption of crypts, responsible for epithelial cell renewal, suggests impaired regenerative capacity in the face of viral assault. Moreover, the inflammation of villi compromises their absorptive function, potentially leading to malabsorption and nutrient deficiencies.

**Figure 5:**
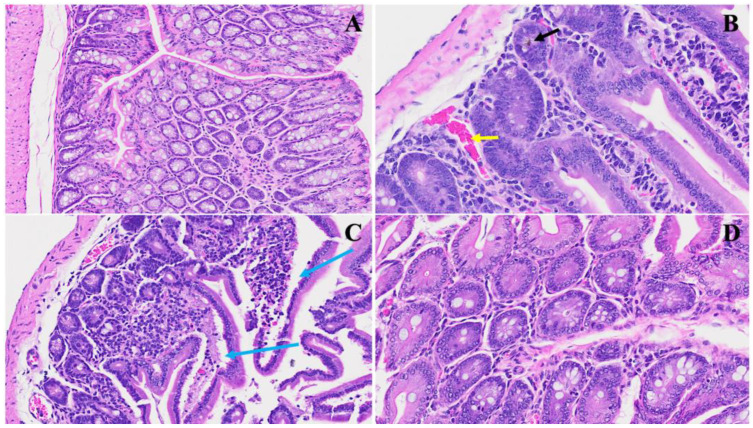
SPK restoration of intestinal architecture post MHV-1 infection. Panel (**A**) displays standard intestinal architecture in a control group. In Panel (**B**), acute inflammatory changes, microthrombi (yellow arrow), and hemosiderin deposition (black arrow) are evident in ileum. Panel (**C**) illustrates acute severe inflammatory changes resulting in villi destruction (blue arrows). Panel (**D**) demonstrates the restoration of these changes following SPK treatment. (H&E, original magnification 66× (**A**–**D**)). (MHV-1 infection alone (*n* = 16), healthy control (*n* = 7), and SPK-treated mice (*n* = 5)).

We noted the potential efficacy of SPK in a novel experimental model of intestinal damage induced by MHV-1 infection. Figure 5B shows acute inflammatory changes accompanied by microthrombi (yellow arrow) and a unique hemosiderin deposition (black arrow). Further, in Panel C, we observe an extent of damage, with severe inflammatory changes leading to villi destruction (blue arrows). Remarkably, Panel D demonstrates the restoration of intestinal architecture following SPK treatment. The resolution of acute inflammatory changes, disappearance of microthrombi, and restoration of villi structures suggest the therapeutic efficacy of SPK in mitigating intestinal damage induced by viral infection.

**Figure 6:**
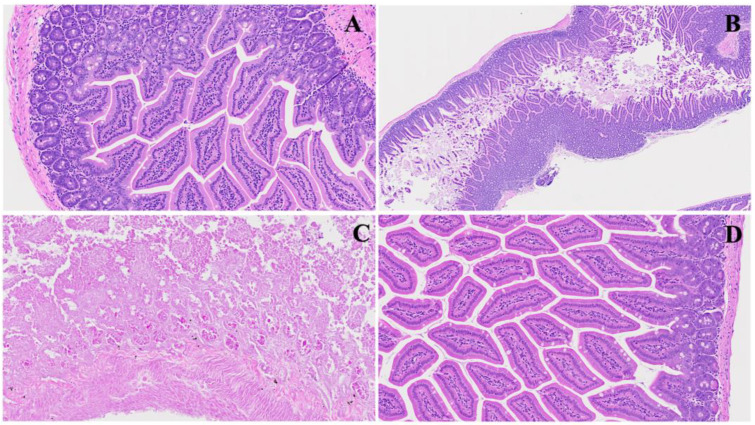
Acute ileum changes post MHV-1 infection. Panel (**A**) illustrates the histology of a normal ileum. In Panel (**B**), acute severe hyperplasia of crypts is observed, accompanied by various stages of inflammatory changes. Panel (**C**) showcases the massive destruction of one part of the ileum, characterized by the blunting of crypts and villi and the loss of brush borders. Panel (**D**) depicts the restoration of tissue architecture following SPK treatment. (H&E, original magnification 66× (**A**,**D**) and 22× (**B**,**C**)). (MHV-1 infection alone (*n* = 16), healthy control (*n* = 7), and SPK-treated mice (*n* = 5)).

Examining small ileum histopathology following MHV-1 infection sheds light on potential parallels with Crohn’s disease progression. We found, in Panel B of Figure 6, that the acute phase of infection is marked by severe crypt hyperplasia and sloughing of villi, indicative of an aggressive inflammatory response akin to what is observed in Crohn’s disease [23,24]. Panel C portrays a more advanced manifestation, reflecting the destructive nature of the infection on the ileal tissue in one part of the ileum. This stage is typified by extensive destruction, including blunting of crypts and villi and loss of brush borders, reminiscent of the histopathological changes seen in Crohn’s disease, where chronic inflammation leads to significant structural damage and functional impairment. Interestingly, Panel D shows a substantial improvement in the damage that occurred by MHV-1 post-administration of SPK. The crypt–villus structure appears re-established, and brush borders are evident again.

**Figure 7:**
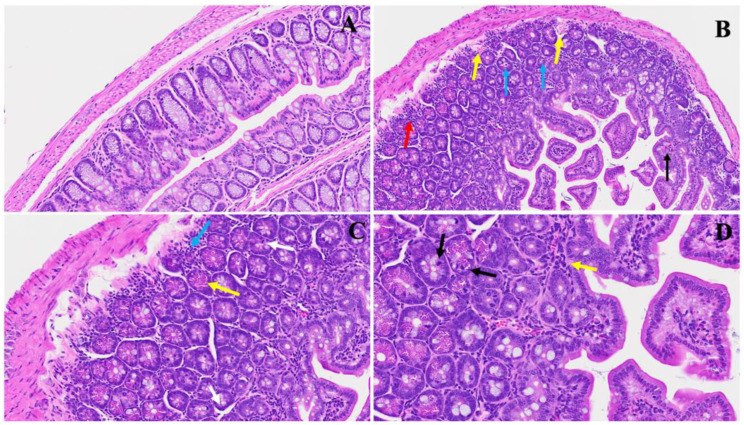
This illustrates the duodenum intestinal changes associated with long COVID. In Panel (**A**), a depiction of standard small intestinal architecture is shown. Panel (**B**) highlights alterations observed in long COVID, including nests of erythrocytosis indicated by yellow arrows, diffused inflammation marked by a red arrow, lymphocyte invasion denoted by blue arrows, along with an increased number of goblet cells, various apoptotic bodies, congestion, and thrombosis indicated by a black arrow. Panel (**C**) exhibits severe inflammatory cellular invasion (blue arrow), accompanied by an increase in Paneth cells (yellow arrows) and the presence of neutrophils (white arrow). Panel (**D**) demonstrates various inflammatory cell infiltrates (yellow arrow) alongside erythrocytosis, with evident apoptotic bodies (black arrows) and an increase in Paneth cells. (H&E, original magnification 66× (**A**–**D**)). (4 MHV-1 infection, 4 healthy control, 4 SPK treated group).

A novel investigation established in our laboratory demonstrated a unique intestinal finding in long COVID. In our experimental model, we found various changes (Figure 7). Panel B shows distinct pathological features; noteworthy are the presence of nests of erythrocytosis denoted by yellow arrows, the diffused inflammation marked by a prominent red arrow, and the infiltration of lymphocytes depicted by blue arrows. Furthermore, an upsurge in goblet cells, various apoptotic bodies, congestion, and thrombosis, indicated by a discernible black arrow, collectively contribute to the potential pathological profile associated with long COVID, as evident in our model. In Panel C, a pronounced inflammatory cellular invasion is apparent, evidenced by an extensive infiltration of inflammatory cells denoted by the blue arrow. Concurrently, an increase in Paneth cells, highlighted by yellow arrows, and the presence of neutrophils, indicated by a white arrow, suggest an altered immune response in the affected tissue. Panel D further elucidates the complexity of the inflammatory milieu observed in long COVID, with scattered inflammatory cell infiltrates depicted by yellow arrows, accompanied by nests of erythrocytosis and conspicuous apoptotic bodies marked by black arrows. The heightened presence of Paneth cells emphasizes the dynamic interplay between the viral infection and the host immune response.

**Figure 8:**
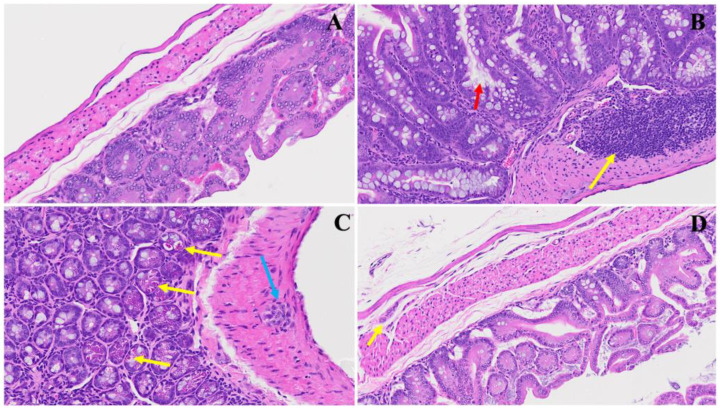
This depicts the SPK effects observed in long COVID in the small intestine (jejunum and ileum). Panel (**A**) presents the standard architecture observed across all intestinal layers. In Panel (**B**), lymphoid hyperplasia is highlighted by the yellow arrow, alongside an increase in goblet cells with large mucus discharges indicated by the red arrow. Panel (**C**) illustrates Auerbach’s plexus (blue arrow), dying Paneth cells showing apoptotic bodies (yellow arrows), and diffused inflammatory cell infiltrates. Panel (**D**) demonstrates the restoration of regular intestinal layers, normalized goblet cell numbers, reduced inflammation, and the return of average Paneth cell counts. Additionally, the presence of the submucosal (Meissner’s) plexus and myenteric (Auerbach’s) plexus (yellow arrow) in their normal state is noted. (H&E, original magnification 66× (**A**,**D**) and 22× (**B**,**C**)). (4 MHV-1 infection, 4 healthy control, 4 SPK treated group).

The investigation of SPK effects within the realm of long COVID unveils discernible alterations in the architectural integrity of the intestine. We noticed, in Figure 8B, that prominent deviations emerge, indicative of pathological changes such as lymphoid hyperplasia, as denoted by the yellow arrow, suggesting an augmented mucosal immune response, potentially in response to persistent viral presence. Further, the increase in goblet cells with prominent mucus discharges, highlighted by the red arrow, implies a heightened mucin production, possibly as a protective mechanism against viral invasion and associated inflammation. We also detect, in Panel C, that Auerbach’s plexus, an integral component of the enteric nervous system, is depicted (blue arrow) alongside dying Paneth cells displaying apoptotic bodies (yellow arrows) and diffused inflammatory cell infiltrates. These findings underscore the dysregulated neural regulation and ongoing inflammatory milieu within the affected tissue, which may also trigger further inflammation. Meanwhile, in Panel D, following intervention with SPK, a remarkable restoration of regular intestinal layers is observed. The normalization of goblet cell numbers, reduction in inflammation, and restoration of Paneth cell counts signify the therapeutic efficacy of SPK in mitigating pathological alterations associated with long COVID. Additionally, the preservation of the submucosal (Meissner’s) plexus and myenteric (Auerbach’s) plexus in their normal state validates the capacity of SPK to modulate intestinal responses and restore tissue homeostasis in the context of long COVID.

## 4. Discussion

The investigation delved into the multifaceted impact of viral infections, particularly MHV-1, on the gastrointestinal tract, shedding light on potential parallels with COVID-induced gastrointestinal complications. Notably, we found acute significant intestinal changes akin to inflammatory bowel disease (IBD), including mucosal inflammation, lymphoid hyperplasia, and microthrombus formation, suggesting a complex interplay between viral invasion, immune response dysregulation, and tissue damage. Additionally, we found villus degeneration, enterocyte destruction, and inflammatory cell infiltrates, parallel pathological features observed in conditions like celiac disease and IBD. Furthermore, our investigation extended to long COVID, revealing distinct pathological profiles characterized by inflammatory cell infiltration, altered immune responses, and architectural disruptions in the intestine. The study’s findings deepen our understanding of viral-induced intestinal pathology, offering insights into potential mechanisms underlying COVID-associated gastrointestinal complications and long COVID. We also examined the efficacy of our protein-based medication (SPK) in mitigating intestinal damage and restoring tissue homeostasis; we identified its potential as a promising therapeutic intervention for COVID-induced gastrointestinal complications and long COVID. These findings not only contribute to elucidating the pathogenesis of viral-induced intestinal pathology but also pave the way for developing targeted therapeutic strategies to alleviate gastrointestinal manifestations of viral infections and improve patient outcomes.

In the acute phase of the infection, we have reported, for the first time, the dissemination of coronavirus through the bloodstream to various organs, including the intestine. This builds upon previously documented findings of viral load detection in the blood and the presence of the virus within multiple organs [14,15,16,17,18,19]. We also reported the presence of the virus and viral particles in the brains of infants born to mothers infected with SARS-CoV-2 [21]. Indeed, viral particles, specifically nucleocapsids, have been identified within the nuclear membrane. Viral particles also sequestrated in the nucleus suggest possible adverse effects in both acute and long COVID, as studies have shown cell injury when exposed to SARS-CoV-2 viral particles in vitro [10,12]. 

Since the onset of the COVID-19 pandemic in 2020, a comprehensive examination of its clinical manifestations has unveiled diverse and often enigmatic multiorgan pathological changes and complications. Our prior study documented diverse clinical manifestations and alterations in mice during both the acute and prolonged phases of COVID-19 [15]. Our investigations have focused on elucidating the potential alterations in the intestine, revealing a spectrum of pathological changes in a well-established mouse model of COVID-19. Over time, it is anticipated that various intestinal disorders may emerge as sequelae of COVID-19. These potential disorders encompass but are not limited to IBD, celiac, colonic malignancy, etc. [23,24,25,26]. The etiology of these disorders is likely multifactorial, involving intricate interactions between the virus and the host’s genes. While the virus could potentially trigger gastrointestinal disorders, its direct role in causing genetic mutations leading to these conditions is still under study. Understanding the complexity of the SARS-CoV-2 virus and its potential involvement in gastrointestinal disorders is crucial. This highlights the importance of ongoing research to clarify how the virus may affect genetic pathways and contribute to the development of these disorders. 

A large cohort study accomplished by US Department of Veterans Affairs national health care databases of 154,068 short-term COVID-19 survivors as well as 5,859,621 patients of long COVID (1-year post-infection). The study showed the development of various gastrointestinal manifestations among these individuals, including IBD, irritable bowel syndrome, dyspepsia, chronic diarrhea, intestinal angina, gastric acid reflux disorder, abdominal pain, and other liver and pancreatic diseases [27]. Cheung et al. found that 48% of COVID-19 patients have fecal shedding of SARS-CoV-2 RNA, which suggests intestinal epithelial cell infection [28]. Xiao et al. identified the SARS-CoV-2 N protein in the intestinal epithelium, which favors intestinal tropism [29]. 

The significant expression of angiotensin-converting enzyme 2 (ACE2) and transmembrane protease serine subtypes 2/4 (TMPRSS) on the lining of the small intestine’s mucosa links acute SARS-CoV-2 infection with gastrointestinal symptoms such as nausea, vomiting, diarrhea, and abdominal pain, while long term reported symptoms are appetite loss, weight loss, and irritable bowel syndrome, albeit with varying prevalence rates in the context of post-acute sequelae of SARS-CoV-2 infection (PASC) [27,28,29,30]. Various evidence indicates persistent inflammation and likely induction of autoimmunity in certain PASC patients, possibly influenced by the continued presence of the virus or viral proteins in multiple organs, including the gastrointestinal tract [27,30,31,32]. Elevated levels of cytokines and specific immune cell types in the blood of PASC individuals suggest a prolonged inflammatory response, highlighting the intricate interplay between viral persistence, immune dysregulation, and possible autoimmune reactions in PASC development [27,33]. Furthermore, in PASC, reactivation of latent viruses like Epstein–Barr virus or cytomegalovirus may contribute to the pathogenesis due to either direct invasion and/or immune mediation (T cells and cytokines), particularly when autoantibodies targeting IFNα2 may impede the body’s immune response and correlate with increased levels of inflammatory cytokines [27,28,29,30]. In addition to the overarching considerations previously outlined, distinctive characteristics of the gastrointestinal mucosal immune system could play a pivotal role in the pathophysiology of gastrointestinal PASC. Specific mechanisms potentially contributing to gastrointestinal PASC encompass intestinal dysbiosis, maladaptive neuro-immune interactions, viral persistence, and abnormal immune activation within the gastrointestinal tract [27,31,34].

Investigations have also examined the correlation between the gut microbiome and PASC [34]. Liu et al. conducted a study wherein they analyzed the fecal microbiome composition using shotgun metagenomic sequencing in a prospective cohort of 106 patients with varying severity of COVID-19, tracking them from admission up to 6 months [34]. The authors found a significant reduction in microbial diversity and distinct gut microbiome profiles associated with PASC [34].

Given the high incidence of motility-related disorders in gastrointestinal PASC, it is essential to consider post-infectious neuro-immune-related disorders in the disease’s pathogenesis [31,33,34,35]. The pathophysiology of these post-infectious gut–brain disorders remains unclear, hampered by limited studies of varying sizes and different time points assessed post-infection. Proposed concepts include microbial dysbiosis, increased intestinal permeability, and low-grade activation of the intestinal immune system [27,34,36]. Further, Lai et al. found communications between gut sensory neurons (nociceptors), gut epithelial cells, and microorganisms, which collectively synchronize mucosal host defense mechanisms [34,36]. Furthermore, macrophages residing in the muscularis propria, closely interacting with enteric neurons, adopt tissue-protective roles that inhibit neuronal damage after infection [34,35,36,37,38]. 

We previously reported the therapeutic advantage of SPK in the liver, skin, brain, and renal [15,16,17,18,19,39]. We also found that SPK downregulates TGF-β in the kidney, which ultimately improved renal histopathology post-SPK treatment. These findings highlighted the potential therapeutic benefits of SPK treatment, emphasizing its role in mitigating specifically intestinal damage and restoring tissue homeostasis, which is particularly evident in restoring regular histopathological changes and normalizing inflammatory responses. Moreover, SPK intervention demonstrated promising outcomes in preserving intestinal integrity, reducing inflammation, and modulating immune responses, emphasizing its potential as a therapeutic avenue for addressing long COVID sequelae of viral infections on the gastrointestinal tract. 

TGF-β initiates an intracellular signaling cascade involving proteins like Smad proteins that transduce the TGF-β signal from the cell membrane to the nucleus [40,41,42,43,44]. In the nucleus, Smad proteins and other transcription factors regulate the expression of genes involved in collagen synthesis, including genes that encode various types of collagens, the principal component of the extracellular matrix [40,41,42,43,44]. It found that TGF-β acts as a negative regulator of mucosal inflammation and indicates that defective production/activity of TGF-β can potentially lead to the development of severe IBD [44]. Further, it was discovered that TGF-β1 expression increased, but the TGF-β1 mediated immunosuppression significantly decreased due to high Smad7 (considered an inhibitory signal to TGF-β1) [44]. Additionally, knocking out Smad7 leads to restoring TGF-β1 activity and reducing the inflammation associated with IBD [44]. 

## 5. Conclusions

In conclusion, our investigation into histopathological changes in the small intestine following MHV-1 infection in murine models unveils significant parallels with IBD, celiac disease. Noteworthy observations encompass mucosal inflammation, lymphoid hyperplasia, goblet cell hyperplasia, and immune cell infiltration, resembling pathological features seen in IBD. Furthermore, MHV-1 infection induces villous atrophy, altered epithelial integrity, and inflammatory responses akin to celiac disease. Encouragingly, SPK treatment effectively mitigates intestinal damage caused by MHV-1 infection, restoring tissue architecture and ameliorating inflammatory responses. Our exploration of long COVID reveals intricate inflammatory profiles, underscoring SPK’s potential to modulate intestinal responses and restore tissue homeostasis. These findings shed light on the pathogenesis of COVID-induced gastrointestinal complications and offer insights for targeted therapeutic strategies. The promising efficacy of SPK as a therapeutic intervention to mitigate long COVID intestinal alterations initiated by SARS-CoV-2 provides hope in addressing the enduring effects of the pandemic.

## Data Availability

The data presented in this study are available upon request from the corresponding author. However, due to the University of Miami Miller School of Medicine’s privacy policy, they are not publicly available.
